# Viral load suppression in HIV-infected adolescents in cameroon: towards achieving the UNAIDS 95% viral suppression target

**DOI:** 10.1186/s12887-023-03943-0

**Published:** 2023-03-15

**Authors:** Armando B. D. Djiyou, Calixte Ida Penda, Yoann Madec, Grace Dalle Ngondi, Astrid Moukoko, Marie Varloteaux, Laure-Amélie de Monteynard, Cecile Moins, Carole Else Eboumbou Moukoko, Avelin F. Aghokeng

**Affiliations:** 1 Department of Biological Sciences, Faculty of Medicine and Pharmaceutical Sciences, University of Douala, Douala, Cameroon; 2 Department of Clinical Sciences, Faculty of Medicine and Pharmaceutical Sciences, University of Douala, Douala, Cameroon; 3 Department of Pediatrics and Child Health, General Hospital of Douala, Douala, Cameroon; 4 Epidemiology of emerging diseases, Institut Pasteur, Université de Paris, F-75015 Paris, France; 5 Hôpital Laquintinie, Douala, Cameroun; 6 Agence Nationale de Recherches sur le Sida et les hépatites virales (ANRS | Maladies infectieuses émergentes), Paris, France; 7 Centre Pasteur du Cameroun, Yaoundé, Cameroun; 8 UMR MIVEGEC, IRD, Université de Montpellier, CNRS, IRD, 911, avenue Agropolis, PO. Box 64501, 34394 Montpellier, Montpellier Cedex 5, France

**Keywords:** HIV-AIDS, Antiretroviral treatment, Adolescents, Viral load suppression, Cameroon

## Abstract

**Background:**

Achieving the UNAIDS 95% sustained viral suppression (VS) rate requires considerable global efforts, particularly among adolescents living with HIV (ALHIV) who are often associated with high rates of virological failure (VF). In this study, we prospectively assessed the rate of VS, and the factors associated with VF in a cohort of adolescents followed up according to the WHO guidelines in Cameroon.

**Methods:**

A cross-sectional study was carried out in 2021 among adolescents (aged 10–19 years) receiving ART in the national program in Cameroon. Socio-demographic and clinical data were collected using patients’ medical files and a brief interview with the participant and/or his guardian. Thereafter, a first viral load test (VL1) was performed using the ABBOTT Platform. For adolescents with VL1 > 1000 copies/ml, adherence-enhancing interventions were routinely performed each month for 3 consecutive months, after which a second viral load (VL2) was measured. Adolescents with VL2 > 1000 copies/ml were considered in VF.

**Results:**

Overall, 280 adolescents were enrolled, of whom 89.3% (250/280) acquired HIV infection via mother-to-child transmission. The median age was 16.0 (IQR: 13.0–18.0) years and the median duration on ART was 9.8 (IQR: 5.1–12.8) years. Females and males were almost equally represented, as 52.1% (146/280) were female, while 47.9% (134/280) were males (p = 0.47). The VS rate was 88.2% (CI: 83.8-91.7%) overall; 89.0% (CI: 82.0-93.1%) and 88.7% (CI: 81.2-93.0%) in females and males, respectively. Being on second or third-line ART, self-declared suboptimal adherence, and a history of past VF were independently associated with VF.

**Conclusion:**

The high rate of VS we report in this study is welcome in the era of the 95/95/95 UNAIDS goals, and indicates that improving treatment outcomes in this specific and fragile population that represent adolescents in Sub-Saharan Africa is achievable.

**Trial registration:**

20/10/2020 NCT04593979 (https://clinicaltrials.gov/ct2/show/NCT04593979).

In its 2021 report, the UNAIDS highlighted poor treatment outcomes among adolescents. Here, we report a virological suppression rate of 88.2% among adolescents. Our experience can guide future options to improve care and treatment in this key and fragile population.

## Background

The human immunodeficiency virus (HIV) remains a major public health concern, which is amplified by the difficulties in accessing essential HIV prevention, testing, and treatment services due to the COVID-19 pandemic [1]. An estimated 37.7 million people were living with HIV (PLHIV) globally, of which about 1.75 million were adolescents aged 10 to 19, and nearly 90% of them resided in sub-Saharan Africa (SSA) [2]. Besides, HIV is more concerning among children and adolescents, who represent only 5% of PLHIV, but 15% of AIDS-related deaths [3].

In 2014, the Joint United Nations Programme on HIV/AIDS (UNAIDS) adopted ambitious fast-track targets that is by 2020, 90% of people living with HIV know their HIV status, 90% of people who know their status receive treatment, and 90% of people on treatment have suppressed viral load (VL). Recently, this target was increased to 95% by 2025 [4]. The majority of Sub-Saharan African countries are far from achieving the 95-95-95 targets by 2025, only Botswana has achieved all three targets so far in SSA [5]. In December 2021, the scorecard finds that countries in the African region reported 77% of PLHIV are on antiretroviral therapy and 68% have suppressed VL globally [1]. Global data show huge disparities in achieving UNAIDS targets between countries and age groups, especially among adolescents who are still lagging behind in accessing ART [6] and achieving viral suppression (VS) compared to adults [7]. Indeed, only 55% of the estimated 1.75 million HIV-infected adolescents received ART in 2020, with the lowest treatment coverage in West and Central Africa (43%) [6]. However, data about the extent to which adolescents are affected in sub-Saharan Africa are limited. Understanding the magnitude of the problem could help clinicians and policymakers tailor interventions aimed at optimizing adolescent HIV care. A multiregional and retrospective cohort study involving data from individuals initiating ART between January 1, 2010, and December 31, 2019 in 31 countries showed that, HIV-infected adults are approaching the global target of 95% VS, but progress among children and adolescents was much slower, with only 59% of them having achieved VS 3 years after ART initiation [8].

In Cameroon, the HIV prevalence was estimated to be 3.4% in the general population [9]. Based on the Cameroon Population-Based HIV Impact Assessment (CAMPHIA) and the 2019 AIDS impact module of the Spectrum software estimates, among 504,281 HIV-infected patients nationwide, 77% were tested for HIV infection, 62% were receiving ART and 53% achieved VS [9, 10]. Moreover, VL monitoring remains a major challenge in Cameroon, as only 25% of the 504,281 HIV-infected patients nationwide accessed VL in 2019 [10]. A study conducted between October 2016 and August 2017 among 1946 patients on treatment for at least 12 months and followed up mainly in the city capital of Cameroon showed that the VS rate was significantly lower in adolescents (53.3%), compared to adults (81.1%) and even children (75.8%) [11]. A more recent study conducted in 2019, and involving 270 perinatally-infected adolescents followed up in two urban and two rural health facilities in the Center region of Cameroon reported up to 66% of VS [12].

In this study, we prospectively assessed the rate of VS and the factors associated with virological failure (VF) in a cohort of adolescents living with HIV (ALHIV) on ART in Cameroon, followed up according to the WHO guidelines.

## Materials and methods

### Study setting, populations, and procedures

The study was a cross-sectional assessment that we conducted from February to September 2021. Eligible participants were adolescents, aged 10 to 19 years, receiving antiretroviral therapy (ART), followed up and monitored at the HIV/AIDS treatment center of the Laquintinie Hospital of Douala (LHD), a urban hospital setting in Douala, Cameroon. This HIV treatment center is the second-largest HIV/AIDS treatment center in the country, where an estimated number of 6000 patients under ART are monitored, among which almost 400 are adolescents. As a public health care clinic, patients are followed up and monitored according to national recommendations, which are mostly based on WHO public health recommendations for resources limited settings [13]. According to national and WHO guidelines, VL monitoring should be carried out six months after ART initiation, then at 12 months and every 12 months thereafter [13]. In case of VL above the threshold defining failure (i.e. ≥1000 copies/mL), adherence counselling and support was provided for a duration of 3 to 4 months following national guidelines, after which a new VL measurement was performed.

All the study participants were recruited in this clinic, using the following inclusion criteria: being infected with HIV-1, aged 10–19 years, on ART for a minimum of six months regardless of the ART regimen, and providing parental informed consent and adolescent assent. Sociodemographic and clinical characteristics were collected in medical records using a standardized case report form. Information regarding the number of missed pills over the last five days and the educational level were obtained after interviewing the participant and/or his parent/guardian. Blood specimens were collected at the inclusion visit for the initial VL testing, called VL1. Participants with VL1 < 1000 copies/ml were considered as VL suppressed according to national and WHO guidelines. For those presenting VL1 ≥ 1000 copies/ml, actions were conducted to reinforce treatment adherence, including therapeutic education and enhanced adherence counseling, and a second VL testing (VL2) was performed three months later from the first testing. Participants with VL2 < 1000 copies/mL were considered virally suppressed, while those with VL2 ≥ 1000 copies/ml were considered in virological failure (VF) and were eligible for treatment line modification.

### Sample collection and viral load testing

Laboratory testing was performed according to routine VL testing procedures at the study site. Briefly, whole blood specimens were collected using Ethylenediaminetetraacetic acid tubes and transferred within an hour to the reference study laboratory, the Retrovirology Laboratory of the LHD. Upon reception, samples were processed by centrifugation and the recovered plasma specimens were stored at -80 °C for further utilization. HIV-1 VL testing was performed using the Abbott *m*2000rt HIV-1 assay (Abbott Molecular, Des Plaines, IL, USA) and following the manufacturer’s instructions. To guarantee the quality of results, the Laquintinie Retrovirology Laboratory is part of an international program that supports and assesses molecular biology laboratories in Africa, the Opp-Era Program (https://chargevirale-oppera.solthis.org/).

### Statistical analysis

Adolescents’ characteristics at enrolment were described and compared between girls and boys, and between those infected via mother-to-child transmission of HIV and the others using a chi-2 test or Fisher exact test for categorical variables, and Wilcoxon non-parametric test for continuous variables. The primary outcome of this study was the proportion of adolescents achieving viral suppression (VS) in our study population. The proportions of VS and VF with the 95% confidence interval was computed and data were summarized using tables. Participants were considered in VF if they had two consecutive plasma HIV-1 VL ≥ 1000 copies/mL within a three months interval, following the WHO guidelines [13]. On the other hand, VS was achieved when a participant had a single VL < 1000 copies/mL, either at inclusion or after the three following up months. Factors associated with VF were identified using logistic regression models. Variables with a p-value < 0.20 in univariate analysis, were considered in the multivariate model. Then a stepwise backward procedure was applied to identify factors independently associated with VF.

### Ethical considerations

This study was conducted in accordance with Declaration of Helsinki and written informed consent and assent was respectively obtained from parents/guardians and adolescents in accordance with the Declaration. In addition, the study was approved by the Cameroon National Ethics Committee for Research and Human Health (2020/09/1293/CE/CNERSH/SP) and the Ministry of Public Health under authorization (130–857/MINSANTE/SG/DROS/CRSPE/BBM). All necessary efforts were done to guarantee patients’ confidentiality throughout the study.

## Results

### Participant’s characteristics

Overall, we recruited 280 adolescents. Girls and boys were almost equally represented as 52.1% (146/280) were female (p = 0.47). The median age was 16.0 (IQR: 13.0–18.0) years, with almost no difference between girls and boys (p: 0.99). The majority of the participants had a secondary educational level, 75.7% (212/280), 21.8% were at primary school, and 2.5% reached the university level. Moreover, 89.3% (250/280) acquired HIV infection via vertical transmission by mother-to-child transmission (MTCT), and 10.7% (30/280) by other routes. As regards to the knowledge of their HIV status, 57.4% (160/280) knew their status, and the others were not fully informed (Table 1).

**Table 1:** Baseline characteristics of the study participants

Characteristics	Overall N = 280	Female N = 146	Male N = 134	P
Age (in years), median (IQR)	16 (13–18)	16 (13–18)	16 (13–18)	0.99
Education level, n (%)				0.22
Primary	61 (21.8)	27 (18.5)	35 (26.1)	
Secondary	212 (75.7)	114 (78.1)	97 (72.4)	
University	7 (2.5)	5 (3.4)	2 (1.5)	
Infection mode, n (%)				0.19
Perinatal	250 (89.3)	127 (87.0)	123 (91.8)	
Other ^a^	30 (10.7)	19 (13.0)	11 (8.2)	
Full disclosure of HIV status, n (%)				0.70
Yes	160 (57.4)	85 (58.2)	75 (56.0)	
No	120 (42.6)	61 (41.8)	59 (44.0)	
Treatment line, n (%)				0.99
On first-line ART	147 (52.5)	77 (52.7)	70 (52.2)	
On second/third-line ART ^b^	133 (47.5)	69 (47.3)	64 (47.8)	
Current ART regimen, n (%)				0.99
TDF-3TC-EFV	109 (38.9)	58 (39.7)	51 (38.1)	
ABC-3TC-EFV	30 (10.7)	16 (11.0)	14 (10.4)	
TDF-3TC-LPV/r	85 (30.4)	44 (30.1)	41 (30.6)	
ABC-3TC-LPV/r	30 (10.7)	15 (10.3)	15 (11.2)	
Other ^c^	26 (9.3)	13 (8.9)	13 (9.7)	
Time since ART initiation (years), Median (IQR)	9.8 (5.1–12.8)	9.0 (5.1–12.3)	10.1 (5.1–12.9)	0.31
Access to VL in the last 12 months, n (%)	203 (72.5)	113 (77.4)	90 (67.2)	0.06

^a^Other modes of transmission were: unknown (n = 18), sexual transmission (n = 9) and blood transfusion (n = 3)

^b^Two patients were on third-line regimen (one male and one female)

^c^Other ART at enrolment were: TDF-3TC-ATV/r (n = 15); TDF-3TC- DTG (n = 7); AZT-3TC- NVP (n = 2); TDF-3TC- DTG-LPV/r (n = 1); ABC-DTG-DRV/r (n = 1)

ART, antiretroviral treatment; AZT, zidovudine; ABC, abacavir; 3TC, lamivudine; EFV, efavirenz; TDF, tenofovir; LPV/r, lopinavir boosted with ritonavir

Of the HIV treatment, 52.5% (147/280) of the adolescents were on a first-line regimen, 46.8% (131/280) were on a second-line, and 2 children were receiving a third-line ART. Current ART regimens included Tenofovir/Abacavir + Lamivudine + Efavirenz for first-line treatment, and Tenofovir/Abacavir + Lamivudine + Lopinavir boosted by ritonavir for second-line regimens. One-half of the study participants were receiving ART for almost 10 years, with the median duration on ART significantly longer (p < 0.001) among perinatally-infected adolescents (10.5; IQR: 5.9–13.2) years compared to the others (3.9; IQR: 1.8–6.9) years. Regarding the routine access to virological monitoring, 72.5% (203/280) had at least one VL assessment conducted in the last 12 months before study initiation (Table 1).

### Virological outcome and associated factors

The first VL assessment was successfully conducted for 279 out of 280 participants recruited. One result was invalid at multiple attempts and was excluded. Out of these 279 valid results, 59 were ≥ 1000 copies/mL. After three months of enhancing adherence to ART through therapeutic education, a VL test was performed for 57/59 of these adolescents to confirm VF and two adolescents were lost to follow-up. For these VL confirmations, 24 out of 57 results were < 1000 copies/ml. The 33 remaining results were ≥ 1000 copies/mL, corresponding to 11.8% of the entire study population. Therefore, the overall virological suppression rate was 88.2% (CI: 83.8-91.7%); with no difference according to the gender, 89.0% (CI: 82.0-93.1%) for girls and 88.7% (CI: 81.2-93.0%) for boys.

Factors independently and significantly associated with higher odds of VF were being on second or third-line ART (p = 0.07), self-declared suboptimal adherence (p = 0.043), and having received a previous VL result ≥ 1000 copies/mL in the last 12 months (p = 0.014). Conversely, gender, age groups, and duration on ART or infection route were not associated with failure. When defining failure on the 1st VL ≥ 1000 copies/mL only, results were very similar. Moreover, among adolescents with an initial detectable VL ≥ 1000 copies/mL, our results revealed that those receiving ART for 12 years or more are more likely to maintain a detectable VL ≥ 1000 copies/mL compared to those who have been treated for a shorter time (Table 2).

**Table 2:** Factors associated with virological failure in our study population

Characteristics	All adolescents included in the study							Adolescents with an initial viral load ≥ 1000 copies/mL			
	N = 279	Failure	Crude OR (95% CI)	P	Adj. OR (95% CI)	P		N = 59	Failure	Crude OR (95% CI)	P
Gender Male Female	133 146	16 (12.0) 17 (11.6)	1 0.96 (0.47–1.99)	0.92				27 32	16 (59.3) 17 (53.3)	1 0.78 (0.28–2.19)	0.64
Age (in years) ≤ 12 13–15 ≥ 16	58 79 142	7 (12.1) 9 (11.4) 17 (12.0)	1 0.94 (0.33–2.68) 0.99 (0.39–2.53)	0.99				14 18 27	7 (50.0) 9 (50.0) 17 (63.0)	1 1.00 (0.25–4.04) 1.70 (0.46–6.28)	0.61
Infection mode Perinatal Other	250 29	30 (12.0) 3 (10.3)	1 0.85 (0.24–2.97)	0.79				53 6	30 (56.6) 3 (50.0)	1 0.77 (0.14–4.15)	0.76
HIV status known No Yes	120 159	13 (10.8) 20 (12.6)	1 1.18 (0.56–2.49)	0.65				28 31	13 (46.4) 20 (64.5)	1 2.10 (0.74–5.97)	0.16
Education level Primary Secondary University	61 211 7	9 (14.7) 24 (11.4) 0	1 0.72 (0.32–1.62) 0.37 (0.02-7.00)	0.64				18 41 -	9 (50.0) 24 (58.5) -	1 1.41 (0.46–4.30) -	0.54
ART line First Second/Third	146 133	9 (6.2) 24 (18.1)	1 3.35 (1.50–7.51)	0.002	1 2.25 (0.93–5.41)	0.07		16 43	9 (56.2) 24 (55.8)	1 0.98 (0.31–3.12)	0.98
Duration on ART (years) ≤ 5 5–10 10–12 > 12	67 74 57 81	5 (7.5) 12 (16.2) 4 (7.2) 12 (14.8)	1 2.40 (0.80–7.22) 0.94 (0.24–3.66) 2.16 (0.72–6.47)	0.19				13 22 10 14	5 (38.5) 12 (54.5) 4 (40.0) 12 (85.7)	1 1.92 (0.47–7.66) 1.07 (0.20–5.77) 9.60 (1.48–62.16)	0.039
Suboptimal adherence at M0 No Yes Unknown	146 124 9	9 (6.2) 22 (17.7) 2 (22.2)	1 3.28 (1.45–7.43) 4.35 (0.79–24.05)	0.008	1 2.90 (1.23–8.81) 3.51 (0.56–22.07)	0.043		17 40 2	9 (52.9) 22 (55.0) 2 (100)	1 1.09 (0.36–3.31) 4.47 (0.19-106.96)	0.65
Suboptimal adherence at M3 No Yes Unknown	/	/	/	/	/	/		23 33 3	8 (34.8) 22 (66.7) 3 (100)	1 3.57 (1.19–10.69) 12.76 (0.59-277.46)	0.038
Previous VL in the last 12 months < 40 40-1000 ≥ 1000 No VL	110 42 51 76	8 (7.3) 4 (9.5) 16 (31.4) 5 (6.6)	1 1.34 (0.39–4.72) 5.83 (2.30-14.79) 0.90 (0.28–2.86)	< 0.001	1 1.02 (0.28–3.75) 3.41 (1.25–9.32) 0.86 (0.61–2.24)	0.014		11 10 27 11	8 (72.7) 4 (40.0) 16 (59.3) 5 (45.5)	1 0.25 (0.04–1.56) 0.55 (0.12–2.53) 0.31 (0.05–1.85)	0.40

ART, antiretroviral treatment; M0, inclusion month; M3, third follow-up month; VL, viral load

## Discussion

In this study, we estimated the rate of VS and the factors associated with VF among HIV-1-infected adolescents followed up in the national ART Program in Cameroon according to the WHO guidelines.

Our results showed a VF rate of 11.8% in HIV-infected adolescents, i.e. virological suppression of 88.2%, close to the UNAIDS target of 95% [4]. This low rate of VF is rarely observed compared to other studies conducted in sub-Saharan Africa, probably due to the promptness of the systematic intervention of therapeutic education to improve adherence to ART [14–17]. A cross-sectional study conducted in Ethiopia in 2019, involving 9386 HIV-infected adolescents on ART for at least 6 months, reported a failure rate of 26% [18]. Other previous studies conducted in Kenya (908 participants) and Uganda (567 participants), reported higher rates of VF, 20% and 31%, respectively [19, 20]. The two previously mentioned studies on adolescents that found VS rates of 53.3% and 66% defined VF as a single VL ≥ 1000 copies/mL. However, in this study, the fact that VF was declared after a second VL ≥ 1000 copies/mL following therapeutic education and enhanced adherence counseling have significantly contributed to increase VS rate in our study. Indeed, the fact that up to 26 of the 57 (45.6%) ALHIV with an initial detectable VL ≥ 1000 copies/mL achieved viral suppression at re-testing is a proof that enhance adherence counseling is a critical part of the monitoring of ALHIV and must be widely implemented in our settings.

The high rate of VS observed in this study could be linked to the effort to implement differentiated health service delivery in our setting. Programs that make use of social workers, peer support and training on pediatric disclosure have reported better virological outcomes in HIV-infected children [21] and adolescents [22]. In our study settings, the fact that extended service hours on Wednesday (late afternoon) were offered to adolescents, along with the provision of games and television, have made clinics more adolescent-friendly and therefore improved retention in care and VS as suggested in a previous study in South Africa [23]. Furthermore, the implementation of interventions such as keeping a list of those who had not achieved VS using a high VL register and linking each adolescent to a dedicated case manager offering social, psychological and sometimes financial support to the adolescent and his family have facilitated close monitoring of those failing ART. Although we didn’t directly assess the impact of these various interventions in our cohort of ALHIV, we assume they played a role in the achievement of VS in our study population as recently demonstrated in countries facing similar challenges [24, 25]. Further evaluation studies are therefore needed in our context to better assess the impact of those interventions on treatment outcome, thus allowing to quickly extend their implementation to other programs with poorer VS levels.

Our study also revealed that the odd of VF was higher among ALHIV receiving a second- or third-line ART, with self-declared suboptimal adherence, and having received a previous detectable VL result ≥ 1000 copies/mL in the last 12 months. These findings are particularly concerning because it means that switching ALHIV to a second- or even third-line ART did not improve VS, probably because barriers to adherence were not sufficiently addressed before switching to other regimens. Indeed, adolescence is a period in life during which major changes occur, and HIV-infected adolescents may face issues with the diseases and ART leading to increased rates of VF when not correctly addressed [18, 19]. A study including 12 cohorts of second-line antiretroviral treatment, representing 928 HIV-infected children and adolescents, showed that virological outcomes were 3-fold poorer among adolescents compared to children [26]. Recent studies in South Africa [18], Uganda [19] and Kenya [20] further showed that HIV-infected adolescents receiving a second-line regimen are more likely to experience VF than those on first-line ART. This could be due to the fact that second-line regimens are more complex than first-line regimens, are often twice daily regimens and have more adverse side effects than first-line regimens, hence negatively affecting adherence to ART [18]. Considering the history of poor adherence behavior, it is also possible that in these patients, adherence problems persist even after switching to second-line ART. Therefore, it is crucial to identify and address adherence difficulties while changing ART regimen.

Our study was conducted in a particular context, since the study started almost one year after the onset of the first COVID-19 cases in Africa. Indeed, COVID-19–related stay-at-home orders have prevented patients from attending their routine visits and travel restrictions have affected essential HIV services worldwide [27], including the provision of antiretroviral drugs and reagents for HIV testing and VL. This may partially explain why up to 27.2% (76/280) of ALHIV included in this study did not receive a VL test during the last 12 months. According to national and WHO guidelines, VL monitoring should be carried out six months after ART initiation, then at 12 months and every 12 months thereafter [13]. Given the fact that this study was done in the health facility hosting the reference laboratory in charge of testing VL samples coming from other health facilities located in the Littoral Region of Cameroon, one can expect the situation to be worse in other regions with less access to laboratory infrastructures. In Uganda for instance, COVID-19 has led to a reduction of VL coverage from 96 to 85% between December 2019 and June 2020 [28, 29]. Consequently, the impact of the COVID-19 pandemic on the provision of VL testing could not be excluded in our context knowing that laboratory staff members and equipment continue to be shared between the HIV and COVID-19 responses [29].

This study has three major limitations which should be considered when interpreting the findings. Firstly, the lack of genotyping resistance testing has limited our understanding of the reasons of VF in this cohort of adolescents. Secondly, the study was conducted in one health facility located in an urban area and could not reflect the situation nationwide. Thirdly, in our context marked by a low ART coverage among adolescents [6], this study has estimated VS rate only among ALHIV receiving treatment, and could not be extrapolated to all adolescents affected by HIV in the country.

## Conclusion

We found a high rate (88.2%) of VL suppression among HIV-infected adolescents followed up according to national and WHO recommendations in Cameroon, almost meeting the 2020 UNAIDS/WHO goal of 95% VS. These results support the achievability of this goal in challenging contexts as in Sub-Sharan Africa, provided that necessary actions are in place to improve access to ART and its routine monitoring in this population. However, because continuous access to optimized ART, VL testing, retention in care services and psychosocial support in resource-limited setting remains challenging in Cameroon and in other RLCs, we should still be cautioned on the high risks that virological failure and drug resistance represent in these settings. Particular attention should be given to adolescents with past history of VF, sub-optimal adherence or receiving second- or third-line regimens.

## Data Availability

The datasets used and/or analyzed during the current study are available from the corresponding author upon reasonable request.

## Abbreviations

XML: Extensible markup language
ALHIV: Adolescents living with HIV
ART: Antiretroviral treatment
COVID-19: Coronavirus disease 2019
HIV: Human immunodeficiency virus
LHD: Laquintinie Hospital of Douala
PLHIV: People Living with HIV
UNAIDS: Joint United Nations Programme on HIV/AIDS
VF: Virological failure
VL: Viral load
VS: Viral suppression
WHO: World Health Organization
